# Reports of Lunatic Asylums

**Published:** 1851-05

**Authors:** 


### ARTICLE III.

*Eighth Annual Report of the Managers of the State Lunatic Asylum of the State of New York, made to the Legislature Feb. 27th* 1851.

*Report of the Pennsylvania Hospital for the Insane, for the year* 1850. By Thomas S. Kirkbride, M. D., Physician to the Institution.

*Annual Report of the Commissioners and Medical Superintendant of the Indiana Hospital for the Insane.*

*Report of the Joint Committee of the Legislature of Indiana appointed to investigate certain charges against Officers of the Hospital for the Insane.*

The New York State Lunatic Asylum situated at Utica, is the largest institution of the kind in America, though much smaller than several European institutions of the kind. During the past year it has had under treatment 816 patients, of whom 171 were discharged cured, 8 much improved, 49 improved, 108 unimproved, 51 died, and 429 remained in the Asylum at the date of the report. Since the Institution was opened in 1843 it had received up to Nov. 20th 1850, 2743 patients, of whom 1188 had been discharged cured, 468 improved, 328 unimproved and 320 had died. The Institution up to the time of his death was under the management of the distinguished and talented Amariah Brigham, M. D. Since the eighth of December 1849, the Institution has been under the superintendence of N. D. Benedict, M. D., who has from that time, in the language of the Managers "devoted his best energies, and with very satisfactory success, to the management and treatment of the patients committed to his charge."

The part of the Report before us, prepared by Dr. Benedict is full of interest, and shows a familiarity with the subject of insanity, and the management of such institutions that could only be the result of industry and devotion to the subject.

The Pennsylvania Hospital for the Insane is a richly endowed institution, having become so by the rise of property in Philadelphia, around the old Pine Street Institution, of which it is a branch, and by the liberal donations of its friends.

We are rejoiced by the prosperity that has attended it since it has been in operation, now ten years. This success has in a great measure been owing to the good judgment, zeal and devoted attention of Dr. Kirkbridge, under whose care it has been during the whole period. It has generally been amongst the foremost institutions of the kind, to adopt the many improvements that have been introduced in the arrangement of hospitals, and the management of the insane, and now has the most elegantly fitted apartments to be found in our country. Persons used to style in living need style in the treatment of their Mental derangements and hence it is an excellent plan to have a few places in the country that can afford such accommodations. Those seeking such care may be assured of every comfort consistent with insanity in this establishment, as well as the attentions of one of the very best men in our profession as their physician.

During tbe past year there have been in the Hospital 428 patients, of whom 106 were discharged cured, 20 much improved, 41 improved, 21 stationary, and 27 died ; leaving 213 in the Institution at the date of the report, the close of the year.

Having spent several of the best years of our life aiding in getting up, planning and building the Indiana Hospital for the Insane, it is natural that we should take a deep interest in every thing that purtains to its welfare. And as a large number of our readers are directly interested in it, as citizens of the State under whose controul it is managed, and by whose liberality it has been founded and sustained, we shall take occasion to refer to it more at length hereafter, by giving a history of its origin, progress and present condition, with such suggestions as may seem appropriate, in reference to it. We therefore lay this, and the report of the joint committee of the Legislature in reference to charges made against some of its officers aside, for the present, promising to refer to them again.

